# Infectious bursal disease virus affecting interferon regulatory factor 7 signaling through VP3 protein to facilitate viral replication

**DOI:** 10.3389/fcimb.2024.1529159

**Published:** 2025-01-13

**Authors:** Zhiyuan Wang, Yang Chen, Yanyan Chen, Rui Chen, Weiwei Wang, Shichen Hu, Yihai Li, Hongjun Chen, Ping Wei, Xiumiao He

**Affiliations:** ^1^ Guangxi Key Laboratory for Polysaccharide Materials and Modifications, School of Marine Sciences and Biotechnology, Guangxi Minzu University, Nanning, Guangxi, China; ^2^ Institute for Poultry Science and Health, Guangxi University, Nanning, Guangxi, China; ^3^ Shanghai Veterinary Research Institute, Chinese Academy of Agricultural Sciences, Shanghai, China

**Keywords:** infectious bursal disease virus (IBDV), interferon regulatory factor 7 (IRF7), antiviral response, IBDV VP3 protein, viral replication

## Abstract

Interferon regulatory factor 7 (IRF7)-mediated type I interferon antiviral response is crucial for regulating the host following viral infection in chickens. Infectious bursal disease virus (IBDV) is a double-stranded RNA virus that induces immune suppression and high mortality rates in chickens aged 3-6 weeks. Previous studies have shown that IBDV infection antagonizes the type I interferon production to facilitate viral replication in the cell, and IRF7 signaling might play an important role. However, the underlying mechanisms that enable IBDV to block the IRF7 pathway remain unclear. In this study, we found that IRF7 and IFN-β expression were suppressed in DF-1 cells during infection with very virulent IBDV (vvIBDV), but not with attenuated IBDV, while the virus continued to replicate. Overexpression of IRF7 inhibits IBDV replication while knocking down IRF7 promotes IBDV replication. Overexpression of IRF7 couldn’t compensate the IRF7 protein level in vvIBDV-infected cells, which suggested that IRF7 protein was degraded by IBDV infection. By using inhibitors, the degradation of IRF7 was found to be related to the proteasome pathway. Further study revealed that IRF7 was observed to interact and colocalize with the IBDV VP3 protein. Consistent with IBDV infection results, IBDV VP3 protein was observed to inhibit the IRF7-IFN-β expression, affect the degradation of IRF7 protein via proteasome pathway. All these results suggest that the IBDV exploits IRF7 by affecting its expression and proteasome degradation via the viral VP3 protein to facilitate viral replication in the cells. These findings revealed a novel mechanism that IBDV uses to evade host antiviral defense.

## 1 Introduction

Infectious bursal disease (IBD) is a highly contagious and immunosuppressive viral disease caused by infectious bursal disease virus (IBDV), posing a serious threat to the global poultry industry. IBDV is a double-stranded RNA virus belonging to the Birnaviridae family and the Avibirnavirus genus, primarily infecting chicks aged 3-6 weeks. IBDV is classified into two serotypes, serotype 1 and serotype 2. Serotype 1, which is pathogenic to chickens can be further divided into four phenotypes, including classical IBDV (cIBDV) (Cosgrove, 1962), very virulent IBDV (vvIBDV) (Chettle et al., 1989), attenuated IBDV (attIBDV) (Winterfield et al., 1981), antigenic variant IBDV(avIBDV) (Jackwood and Saif, 1987), and recently identified Chinese novel variant IBDV (nvIBDV) (Fan et al., 2019; Wang et al., 2022b). The key pathological manifestation of IBDV infection in chicks is the destruction of the bursa of Fabricius, leading to immune suppression in the flock, increased susceptibility to other pathogens, and ultimately, death (Fan et al., 2022; Wang et al., 2022b). In recent years, as IBDV strains continue to mutate, they have caused significant economic losses to the poultry farming industry (Wang et al., 2022a). However, to date, there is no completed effective method for preventing IBDV infection. Therefore, understanding the innate antiviral immune mechanisms from the perspective of virus-host interactions and enhancing the animals’ antiviral capabilities will be an effective approach to control IBDV infection. However, research on the interactions between the host and IBDV remains insufficient.

Viral infection involves a continuous struggling between the virus and the host (Hage and Rajsbaum, 2019; Qin and Zheng, 2017). During this process, the virus utilizes its proteins to interact with host factors, thereby promoting its replication. Type I Interferons (IFN-α/β) are known to be the critical factors in fighting viral infections, constituting the first line of defense in both animals and humans (Chen et al., 2019). Interferon regulatory factor (IRF) 3 and 7 are two key transcription factors that modulate type I IFN expression upon viral infection and have been extensively researched in mammals (Krishnan et al., 2019; Liu et al., 2024; Ma et al., 2023; Matthews et al., 2014; Šestan et al., 2024; Xu et al., 2015; Zhan et al., 2017). Following virus infection, viral genome is sensed by several pattern recognition receptors (PRRs), including toll like receptors (TLRs), and RIG-Like receptors (RLRs) and cytosolic DNA sensors. These receptors signal through mitochondrial antiviral-signaling protein (MAVS) or stimulator of IFN genes (STING) to activate IKK kinase epsilon (IKKi) and Tank binding kinase 1 (TBK1) (Cui et al., 2014). IKKi and TBK1 phosphorylate IRF3/7, leading to its dimerization, nuclear import, and binding to the promotors to induce expression of IFN-β. IRF3/7 was eventually degraded through the proteasome pathway, thus ending their role in antiviral responses. However, the function of IRF3/7 upon virus infection in chickens is still limited. Studies revealed that IRF3 is genetically deficient in chickens and other avian species (Cheng et al., 2019). Conversely, IRF7 has been confirmed to reconstitute corresponding IFN signaling to respond to both DNA and RNA viral infections in chicken (Cheng et al., 2019; Kim and Zhou, 2015).

Increasing evidence demonstrates that many avian viral and viral proteins evade the host’s IFN response by interfering the IRF7-IFN-α/β signaling pathway. Kim (Kim et al., 2020) observed a significant increase in viral titers when DF-1 cells lacking IRF7 were infected with avian influenza virus, indicated a crucial role of IRF7 in regulating the replication process of avian influenza virus. Gao (Gao et al., 2023) discovered that the σA protein of avian reovirus suppresses the activation of IRF7, thereby inhibiting the production of IFN-I and facilitating the immune evasion process of the virus. In our previous studies, we found that following IBDV infection in specific-pathogen-free (SPF) chickens, the activation of TLR3 continuously increased at 8- and 12-hours post-infection (hpi). However, compared to 8 hpi, the expression of IRF7 in the TLR pathway was downregulated at 12 hpi (Chen et al., 2022). Ouyang’s group (Ouyang et al., 2018) found that MicroRNAs (miRNAs) gga-miR-142-5p reduced the expression of the chMDA5 protein, promoting IBDV replication via an IRF7-dependent pathway in DT40 cells. Therefore, IRF7 is speculated to play an important role in the pathogenic mechanism of IBDV. The aim of this study was to investigate the specific regulation effects of IBDV infection on the IRF7 signaling pathway and to elucidate the underlying mechanism. The findings will provide a theoretical foundation for understanding the role of IRF7 in the antiviral immune response following IBDV infection.

## 2 Materials and methods

### 2.1 Viruses, cells and antibodies

The vvIBDV strain used in this study was NN1172 starin which was isolated and identified by our group (He et al., 2014). The attenuated virulent IBDV strain was a commonly used commercial attenuated live vaccine strain B87 which was purchased from HLJ Animal-use Biological Products Co., Ltd., Beijing, China. Dulbecco’s modified Eagle’s medium (12100061; Gibco, USA) supplemented with 10% fetal bovine serum (12483020; Gibco, USA) was used to culture chicken fibroblast cell line DF-1 cells. Cells were grown at 37°C and 5% CO_2_. Anti-IBDV VP2 mouse monoclonal antibody (mAb) was prepared in our laboratory. Other antibodies used in our study were anti-IRF7 rabbit polyclonal antibody (pAb) (bs-2994R, BIOSS, China), anti-FLAG mouse mAb (CW0287, CWBIO, China), anti-β-actin mouse mAb (CW0096M, CWBIO, China), anti-His mouse mAb (M20001M, Abmart, China), AbBox Fluor 594-labeled goat anti-rabbit IgG (BD9279, Biodragon, China), FITC-labeled goat anti-mouse IgG (BF05001, Biodragon, China), goat anti-rabbit IgG H&L antibody (CW0103S, CWBIO, China), goat anti-mouse IgG H&L antibody (CW0102S, CWBIO, China). Other reagents were the proteasome inhibitor MG132 (A2585, APEXBIO, China), ubiquitin inhibitor PYR41 (B1492, APEXBIO, China), autophagosome formation inhibitor Wortmannin (A8544, APEXBIO, China), protease inhibitors (CW2200S, CWBIO, China), SYBR Green qPCR Mix (11201ES, YEASEN, China), DAPI Staining Solution (C1005, Beyotime, China).

### 2.2 Plasmid construction

To construct the VP3 expression plasmid, the VP3 gene was amplified using vvIBDV strain NN1172 cDNA and cloned into the p3×FLAG-CMV-14 vector with the Flag fused to its 3’ end to yield VP3-Flag. Plasmid harboring chicken IRF7 (GenBank accession no. KP096419) was constructed by cloning the synthesized sequence into pcDNA3.1 with the His tag fused to the 3’ ends to yield IRF7-His.

### 2.3 IBDV infection

DF-1 cells were seeded onto plates/dishes at a density of 1.2 × 10^5^ cells. Upon reaching approximately 90% confluent, the growth medium was removed. The cell monolayers of each well were then inoculated with 100 µL of serially diluted virus in DMEM without foetal bovine serum (FBS). Following a 1-hour adsorption period at 37°C, the cells were washed twice with PBS and maintained with DMEM containing 1% FBS at 37°C.

### 2.4 Quantitative real-time PCR

Total RNA from the indicated cells was extracted using Trizol (CW0580, CWBIO, China) reagent, and reverse transcriped into cDNA using reverse transcriptase (CW2020M, CWBIO, China). To detect the relative RNA quantities changed fold of target genes (IBDV, IRF7, and IFN-β), the quantitative Real-time PCR was performed using the SYBR qPCR Mix with an Applied Biosystem (Thermo, USA) according to the manufacturer’s instructions. RT-qPCR was performed using the following cycling conditions 93 °C for 3 min, 95°C for 15 s, 60°C for 15 s, and 72°C for 20 s, followed by 40 cycles. The results were analyzed using the 2^-△△ct^ method. The RT-PCR primers were listed in **Table 1**.

**Table 1:** List of primers used in RT-qPCR.

Genes	Direction	Sequence	Product (bp)	Accession no. in GenBank
IBDV	Forward	ACCGGCACCGACAACCTTA	117	FJ615511.1
	Reverse	CCCTGCCTGACCACCACTT		
IRF7	Forward	ACCACATGCAGACAGACTGACACT	146	AF268079
	Reverse	GGAGTGGATGCAAATGCTGCTCTT		
IFN-β	Forward	TTCTCCTGCAACCATCTTC	82	NM001024836.1
	Reverse	GAGGTGGAGCCGTATTCT		
β-actin	Forward	CAACACAGTGCTGTCTGGTGGTA	205	NM_205518.2
	Reverse	ATCGTACTCCTGCTTGCTGATCC		

### 2.5 Enzyme-linked immunosorbent assay

The levels of IFN-β in cells cultures were analyzed using an ELISA kit for chicken IFN-β (HEA222Ga, USCN Life Science, China) according to the manufacturer’s instructions.

### 2.6 Western blot

Cell samples were harvested and the expression of denatured proteins was analyzed using SDS-PAGE. Cells were lyse with RIPA buffer (P0013B, Beyotime, China) containing Protease Inhibitor Cocktail (CW2200S, CWBIO, China). The lysates were mixed with 5 × SDS loading buffer (CW0027S, CWBIO, China), boiled for 10 minutes, and separated on 10% SDS-PAGE gels. Proteins were subsequently transferred onto a nitrocellulose membrane (ISEQ00010, Solarbio, China). The membrane was then blocked in 5% (w/v) skim milk for 2 h, followed by incubation with monoclonal or polyclonal antibodies for 2 h. After being washed three times (10 min each) with TBST, the membrane was incubated with either goat anti-rabbit IgG (H+L) or goat anti-mouse IgG (H+L) antibody for 1 h., the protein blots were finally visualized using the WD-9423BC automatic chemiluminescence imaging system (LIUYI, China) for further analysis.

### 2.7 Transfection

DF-1 cells were seeded in dishes and grown to over 90% confluence, then the cells were transfected with different plasmids or small interfering RNA(siRNA), using the Lipo8000™ transfection reagent (C0533, Beyotime, China) according to the manufacturer’s instructions.

### 2.8 Knockdown of IRF7 by RNA interference

A specific siRNA targeting chIRF7 mRNA was designed by GenePharma (Shanghai,China). The siRNA sequences used in the experiment were as follows: siIRF7 (Sense: GCA CAG AGC UCC GGG ACU UUU; Antisense: AAG UCC CGG AGC UCU GUG CUU), and Non-specific control (Sense: UGU UAA CCA CCG CAU CCU U; Antisense: GGA UGC GUG GUU AAG CAU U). DF-1 cells were transfected with the siRNA. At 6 h after transfection, cells were infected with 1 MOI vvIBDV. Cells were then collected at different time points after infection to evaluate the knockdown efficiency of IRF7 by Western blot, replication level of IBDV based viral load detection by RT-qPCR and VP2 protein level by Western blot.

### 2.9 Inhibitor treatment

DF-1 cells were transfected with p3×FLAG-CMV14-VP3 plasmid or infected with vvIBDV (1 MOI). At 6 h after transfection or 2 h after infection, the DF-1 cells were treated with PYR-41 (a ubiquitin inhibitor), Wortmannin (an autophagy inhibitor), or MG132 (a proteasome inhibitor) for an additional 24 h, and cell lysates were subjected to determination of the protein levels of IRF7 and VP3 or VP2 by Western Blot. In the VP3 transfection experiment, PolyI:C should be added as an immune activator when the inhibitors were added.

### 2.10 Co-immunoprecipitation and immunofluorescent staining

Co-Immunoprecipitation (Co-IP) was conducted to determine whether IBDV-VP3 interacts with IRF7. DF-1 cells were co-transfected with pcDNA3.1-His-IRF7 and p3×FLAG-CMV14-VP3 plasmids. After 48 hours post transfection, Co-IP was performed as follows: cells were washed three times with ice-cold PBS and lysed in 500 μL of RIPA lysis buffers (P0013, Beyotime, China) for 30 min. After 12,000×g centrifugation, the supernatants of cell lysates were incubated with 3 μL anti-Flag mouse mAb or control mouse IgG overnight. Subsequently, 30 μL protein A/G agarose (80104G, Invivogen, FR) was added to the lysate mixture for 6–8 h. The beads were collected by centrifugation at 3,000 ×g for 5 min at 4°C and washed five times with ice-cold PBS.

Immunofluorescent (IF) staining was conducted to determine whether IBDV-VP3 co-localized with IRF7 in the cell. DF-1 cells were co-transfected with p3×FLAG-CMV-14-VP3 and pcDNA3.1-His-IRF7 plasmids. At 36 h after transfection, the cells were fixed with 4% paraformaldehyde for 20 min at room temperature and permeabilized for 15 min with 0.25% Triton X-100. After being blocked with 5% skim milk, cells were incubated with anti-His mouse mAb and anti-FLAG rabbit mAb overnight at 4°C. After being washed three times with PBS, cells were further incubated with FITC-labeled goat anti-mouse IgG and AbBox Fluor 594-labeled goat anti-rabbit IgG secondary antibody at room temperature for 1 h. Cellular nuclei were stained with DAPI for 10 min and viewed with an LAS X laser scanning confocal microscope (Leica, Cologne, Germany).

### 2.11 Statistical analysis

Statistical significance was calculated using the Student’s t-test for individual paired comparisons or one-way ANOVA whenever multiple groups were compared. For individual comparisons of multiple groups, the Student–Newman–Keuls *post-hoc* test was used to calculate p-values. All values are reported as means ± standard errors (SEM). All statistical calculations were performed using Primer of Biostatistics.

## 3 Results

### 3.1 vvIBDV infection suppress IRF7 expression in DF-1 cells

**Figure 1:**
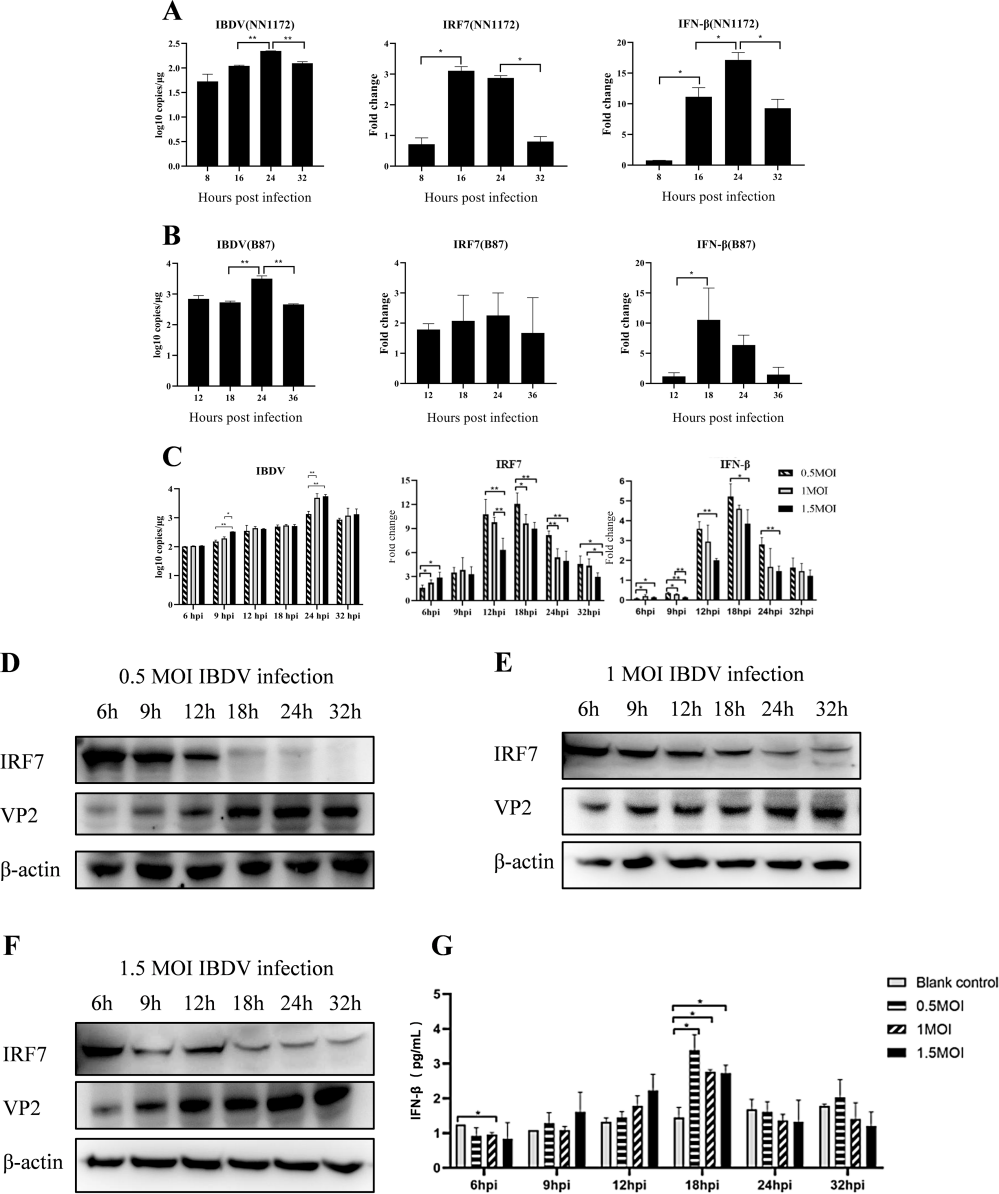
IBDV infection can suppress the expression of IRF7. IBDV viral load, relative expression levels of the IRF7 and IFN-β gene (detected by RT-qPCR) in DF-1 cells infected with 1 MOI vvIBDV NN1172 **(A)** or attenuated IBDV B87 **(B)** at different time points. **(C)** IBDV viral load, relative expression levels of the IRF7 and IFN-β gene in DF-1 cells infected with 0.5, 1, or 1.5 MOI vvIBDV at different time points. **(D-F)** The IRF7 and VP2 protein levels in DF-1 cells (detected by western blot) infected with 0.5, 1, 1.5 MOI vvIBDV at different time point. **(G)** The protein levels of IFN-β at different time points after varying titers of IBDV infection. Three independent experiments were conducted, and the data are presented as the mean ± standard deviation of three replicates from a representative experiment. *p < 0.05; **p < 0.01.

To understand the impact of IBDV infection on IRF7, we infected DF-1 cells with 1 MOI of vvIBDV or attenuated IBDV, respectively. The expression of IRF7 was detected by RT-qPCR, and the results are shown in **Figure 1**. In the vvIBDV-infected group (**Figure 1A**), viral replication levels significantly increased at 24 hpi. The expression levels of IRF7 and IFN-β were significantly upregulated earlier at 16 hpi, but the expression of IRF7 significantly decreased between 24-32 hpi, while the expression level of IFN-β peaked at 24hpi and then down regulated. In the attenuated IBDV-infected group (**Figure 1B**), the viral replication level peaked at 24 hpi, consistent with that in vvIBDV group. However, the mRNA expression of IRF7 showed no significant changes at the detected time points in the attenuated IBDV group. The expression of IFN-β significantly increased only at 18 hpi. The results suggest that vvIBDV suppresses the IRF7 mRNA expression.

In order to further explore the impact of IBDV on IRF7 and IFN-β expression, we infected DF-1 cells with different IBDV titers (0.5, 1 and 1.5 MOI), respectively. RT-qPCR were used to detect IRF7, IFN-β and IBDV VP2 mRNA expression levels. The results, shown in **Figure 1C**, indicate that viral replication peaked at 24 hpi under all the indicated viral titers infection, with highest viral load in 1 and 1.5 MOI group. The mRNA levels of IRF7 were significantly lower in the cells infected by 1 and 1.5 MOI vvIBDV between 12-32 hpi, compared to the levels observed in 0.5 MOI infection group. The mRNA levels of IRF7 significantly lower in 1.5 MOI group than that in 1 MOI group at 12 and 32 hpi. While the expression of IFN-β were the lowest level in the 1.5MOI group at all the detection time point. IRF7 protein and its downstream IFN-β protein levels in the cells were further detected correspondingly by Western Blot and ELISA, respectively. The IRF7 protein was found to be degraded beginning at 18 hpi following all the titers IBDV infection (**Figures 1D-F**), which suggest that the IRF7 protein degradation response to vvIBDV. Consistently, the IFN-β protein peaks at 18 hpi and subsequently declines, with no differences between groups with different dose of IBDV infection (**Figure 1G**). These results suggested that IBDV infection down regulates IRF7 expression and its downstream factor IFN-β while virus replication.

### 3.2 Overexpression of IRF7 inhibits IBDV replication

**Figure 2:**
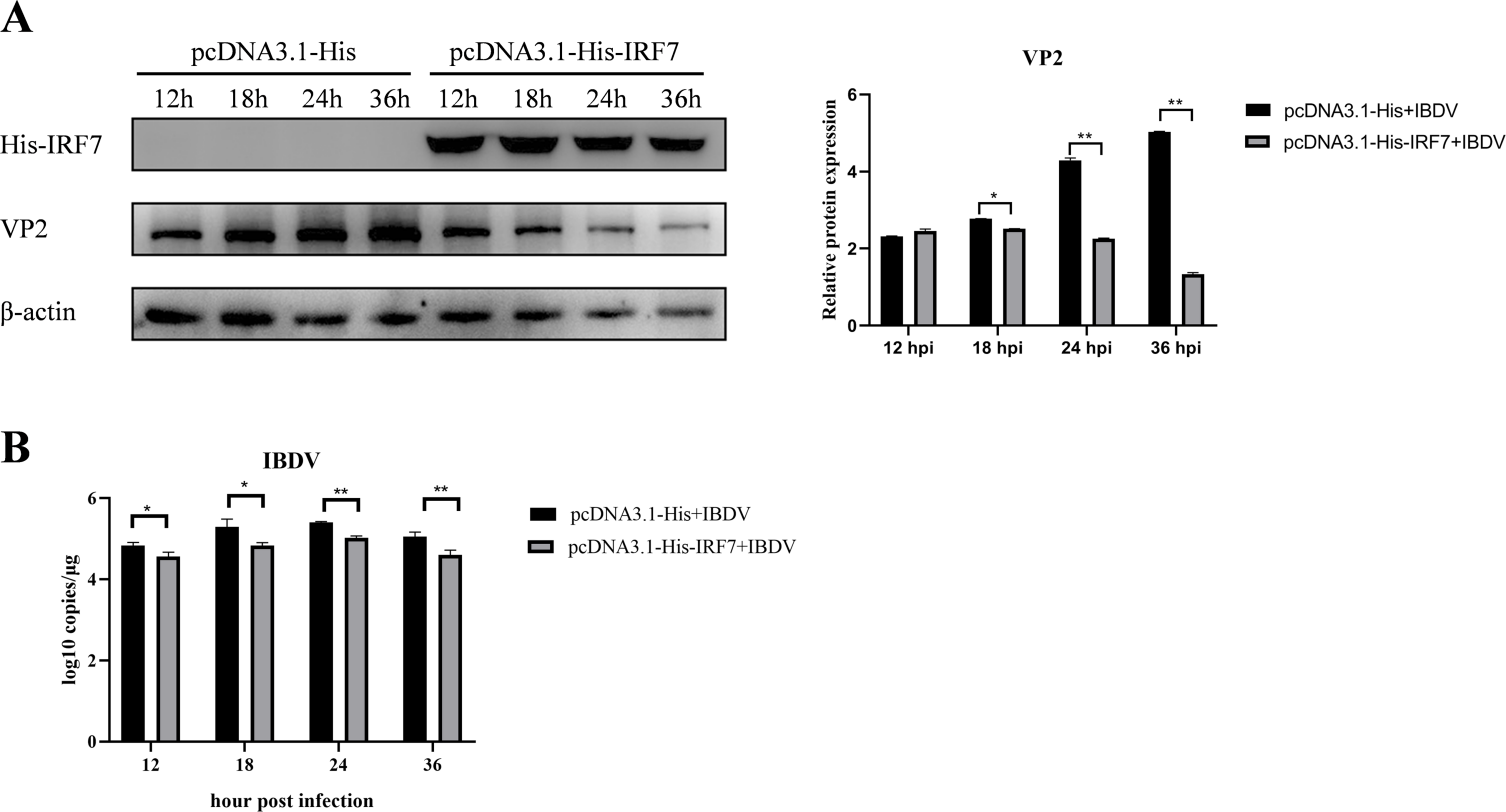
Overexpression of IRF7 inhibits IBDV replication. **(A)** After transfecting DF-1 cells with the pcDNA3.1-His-IRF7 plasmid for 6 hours, cells were infected with 1 MOI vvIBDV. Samples were collected at various time points, and western blot was used to measure the expression levels of IRF7 and IBDV VP2 proteins. Alternatively, relative intensities of VP2 were normalized to β-actin. **(B)** RT-qPCR was employed to measure the IBDV viral load at various time points. Three independent experiments were conducted, and the data are presented as the mean ± standard deviation of three replicates from a representative experiment. *p < 0.05; **p < 0.01.

To further confirm if IRF7 affect IBDV replication, we transfected DF-1 cells with pcDNA3.1-His-IRF7 plasmid and pcDNA3.1-His plasmid, respectively. Following transfection, cells were infected with vvIBDV, and samples were collected at different time points for western blot analysis. As shown in **Figure 2**, in the group transfected with the IRF7 plasmid, the expression of IRF7 protein was significantly higher compared to the empty vector control group as expected. Interestingly, the expression of IBDV VP2 protein was lower in this group than control group (**Figure 2A**). RT-qPCR analysis revealed that cells transfected with pcDNA3.1-His-IRF7 plasmid and then infected with IBDV showed a lower viral load compared to cells transfected with the pcDNA3.1-His plasmid (**Figure 2B**). These results indicated that over expressing of IRF7 is capable of inhibiting IBDV replication.

### 3.3 Knocking down IRF7 promotes IBDV replication

**Figure 3:**
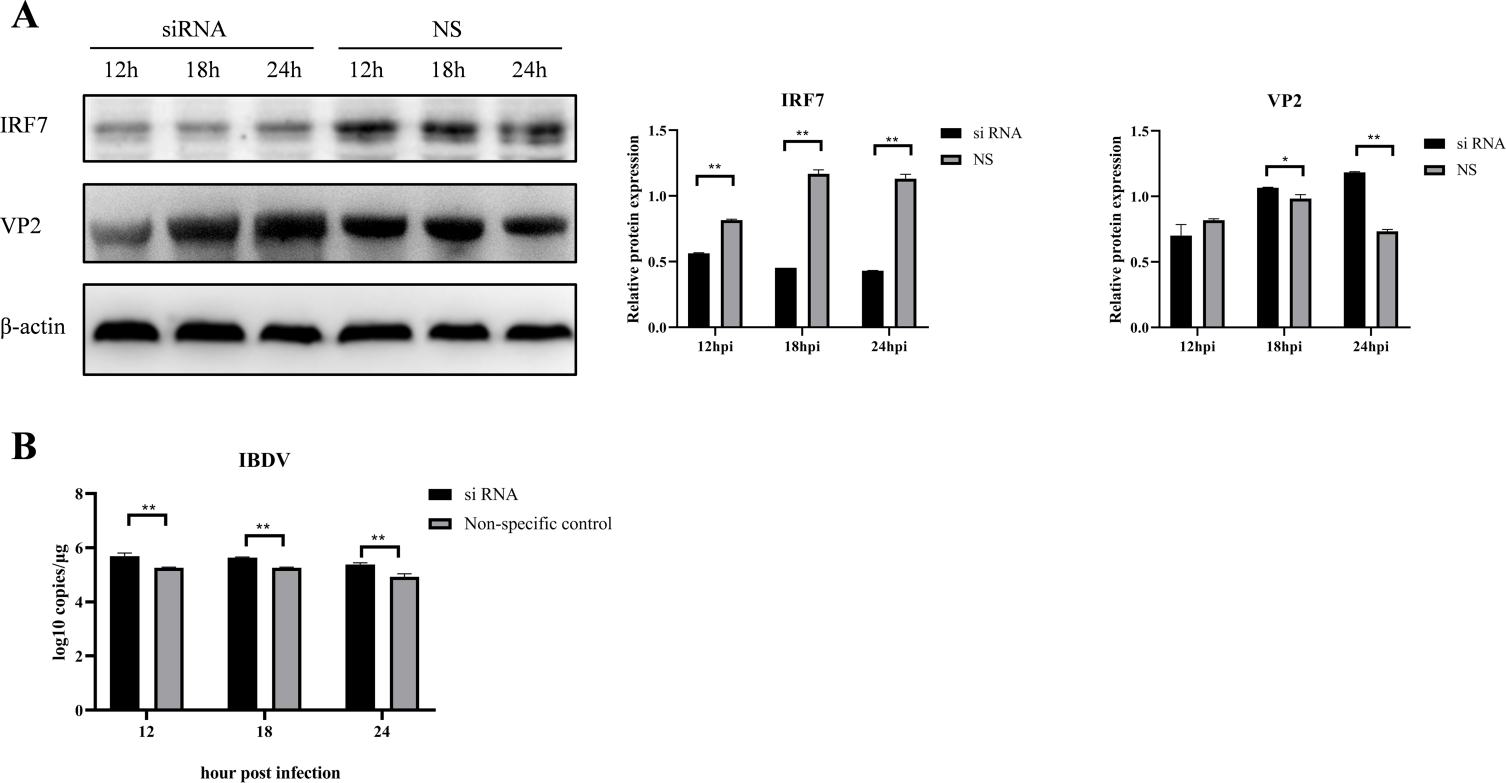
Knocking down IRF7 promotes IBDV replication. **(A)** DF-1 cells were transfected with siRNA targeting IRF7 for 6 hours, followed by infection with 1 MOI vvIBDV. Samples were collected at various time points, and western blot was used to measure the expression levels of IRF7 and IBDV VP2 proteins. Alternatively, relative intensities of IRF7 and VP2 were normalized to β-actin, respectively. **(B)** RT-qPCR was employed to measure the IBDV viral load at various time points. Three independent experiments were conducted, and the data are presented as the mean ± standard deviation of three replicates from a representative experiment. *p < 0.05; **p < 0.01.

To further confirm the affecting of IRF7 on IBDV replication, siRNA targeting IRF7 were used to transfect DF-1 cells, a non-specific (NS) siRNA used as control. Following siRNA transfecting, the cells were infected with vvIBDV, and samples were collected at different time points for western blot analysis. As shown in **Figure 3**, as expected, the expression of IRF7 protein was significantly lower in the cells transfected with IRF7 siRNA compared to the NS control. Consistently, the expression of IBDV VP2 protein was higher in the IRF7 siRNA-transfected group than the NS control group (**Figure 3A**). RT-qPCR analysis further confirmed that cells transfected with IRF7 siRNA had a significantly higher IBDV viral load than the NS control group (**Figure 3B**). These results indicated that knocking down IRF7 promotes IBDV replication.

### 3.4 Overexpression of IRF7 couldn’t inhibit IRF7 degradation in vvIBDV-infected cells

**Figure 4:**
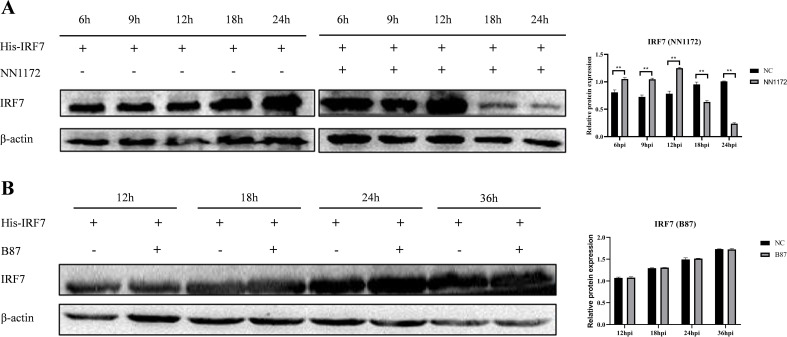
Overexpression of IRF7 couldn’t inhibit IRF7 degradation in vvIBDV-infected cells. **(A)** DF-1 cells were transfected with pcDNA3.1-His-IRF7 and infected with 1 MOI vvIBDV. Samples were collected at 6, 9, 12, 18, and 24 hpi, and the expression of IRF7 protein was detected using western blot. Alternatively, relative intensities of IRF7 were normalized to β-actin. **(B)** DF-1 cells were transfected with pcDNA3.1-His-IRF7 and infected with 1 MOI attenuated IBDV. Samples were collected at 12, 18, 24, and 36 hpi, and the expression of IRF7 protein was detected using western blot. Alternatively, relative intensities of IRF7 were normalized to β-actin. Three independent experiments were conducted, and the data are presented as the mean ± standard deviation of three replicates from a representative experiment. **p < 0.01.

To further confirm if overexpression of IRF7 could compensate the IRF7 protein level when infected by vvIBDV, DF-1 cells were transfected with the pcDNA3.1-His-IRF7 plasmid and then infected with 1 MOI of vvIBDV. The IRF7 protein was detected by western blot. The results showed that the IRF7 protein gradually increased at the early time points, and peaked at 12hpi; however, the expression of IRF7 protein significantly decreased at 18 hpi and afterward, compared to the early time points and uninfected control. As expected, the IRF7 protein gradually increased in the uninfected group until the end of the experiment at 24 hpi (**Figure 4A**), which suggested that the IRF7 protein in the IBDV infected group was degraded.

We also further detected if there were changes in IRF7 protein level in B87-infected group when overexpress the IRF7. DF-1 cells were transfected with pcDNA3.1-His-IRF7 plasmid followed by infection with 1 MOI attenuated IBDV, B87. The western blot analysis results indicated that as the infection time extended, the expression of IRF7 protein gradually increased. However, when comparing the infected group to the uninfected group, the differences in the IRF7 protein production at various time points were not statistically significant (**Figure 4B**) which is different from that in vvIBDV group.

### 3.5 vvIBDV promotes IRF7 degradation through the proteasomal pathway

**Figure 5:**
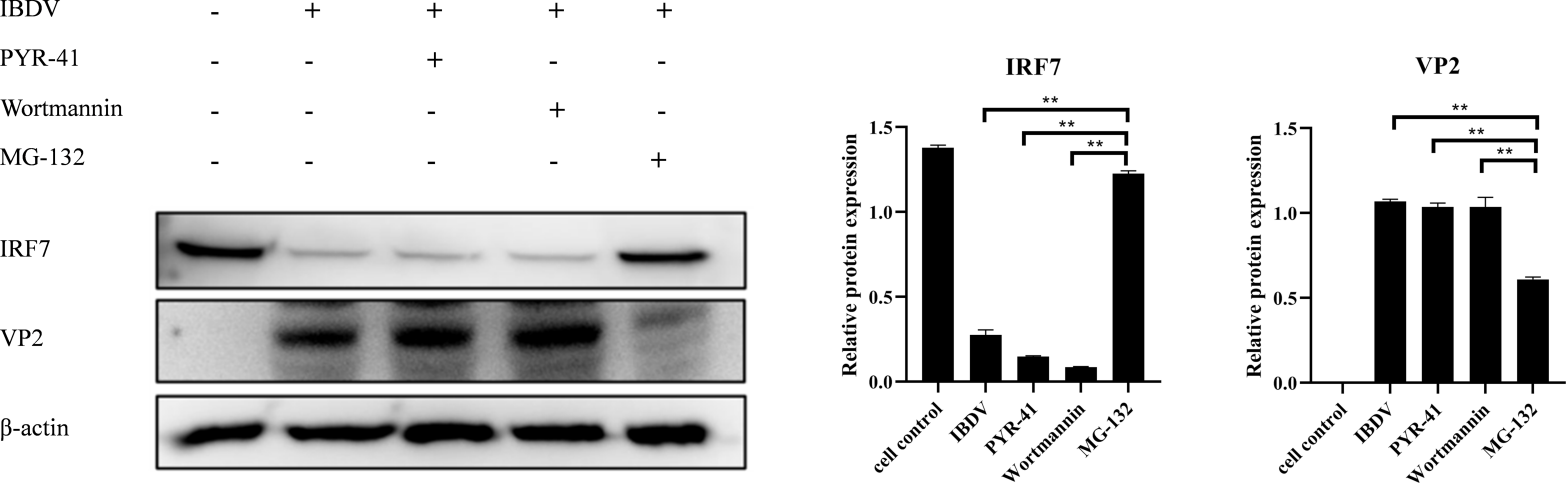
IBDV affects the expression of IRF7 through the proteasomal pathway. In the control group, cells were treated with DMSO, while in the experimental group, cells were treated with different inhibitors (PYR-41, Wortmannin, MG132) after being infected with vvIBDV. Samples were then collected for western blot analysis to measure the expression levels of IRF7 and VP2 proteins. Alternatively, relative intensities of IRF7 and VP2 were normalized to β-actin. Three independent experiments were conducted, and the data are presented as the mean ± standard deviation of three replicates from a representative experiment. **p < 0.01.

To explore which pathway IBDV employs to affect IRF7 degradation, the cells were treated with PYR-41, Wortmannin, or MG132 after infected with 1 MOI of vvIBDV, respectively. IRF7 expression and viral VP2 were detect by western blot. The results are shown in **Figure 5**, the protein level of IRF7 in cells treated with MG132 was significantly higher compared to other inhibitor treated groups, and showed no different with untreated uninfected control. Additionally, the protein level of IBDV VP2 was lower in this group compared to other experimental groups, which indicated lower level of viral replication in the cells treated with proteasome inhibitor MG132. These results suggest that the degradation of IRF7 protein level induced by IBDV infection is mediated through the proteasome pathway.

### 3.6 IBDV VP3 interacts with IRF7

**Figure 6:**
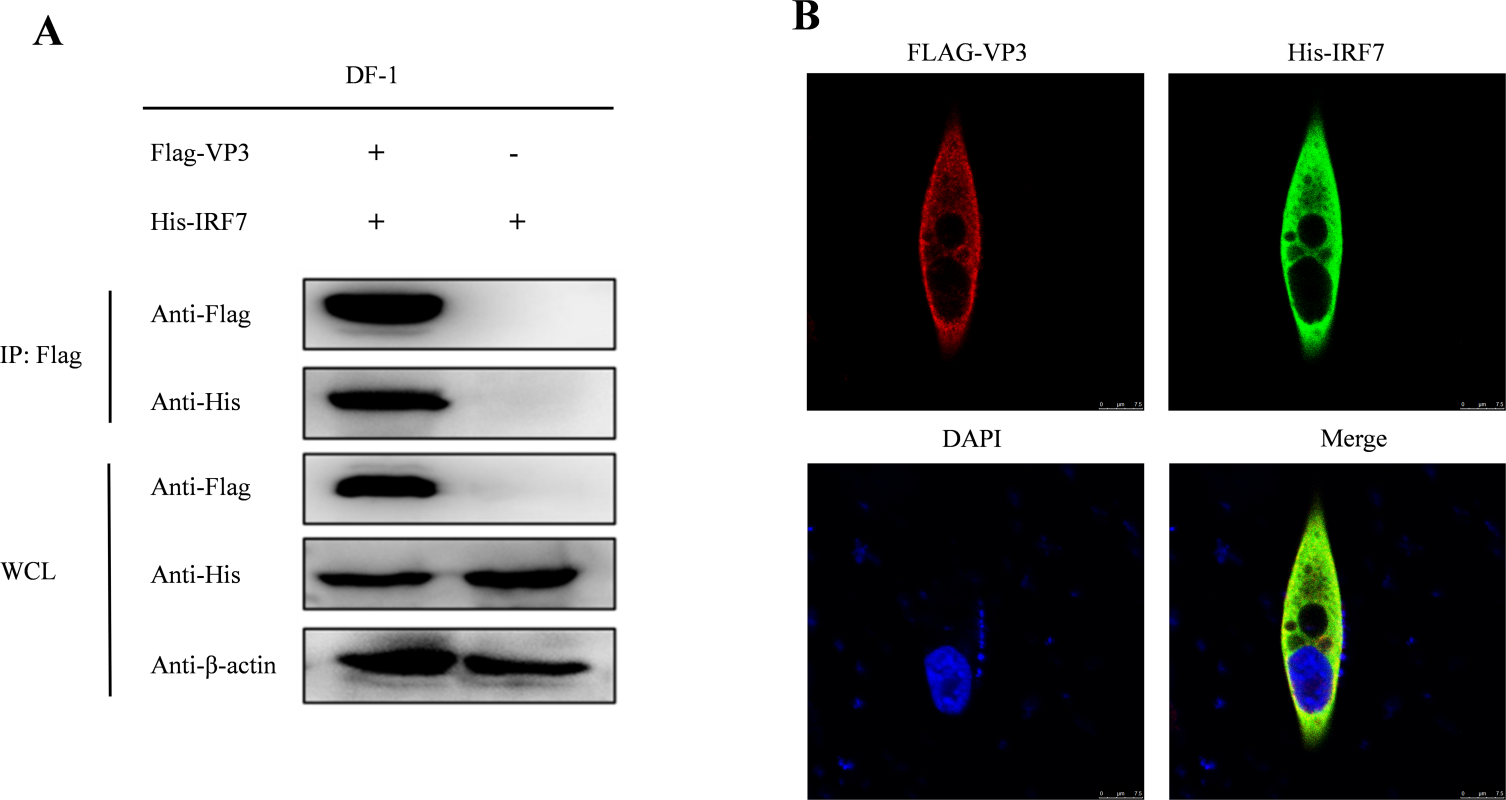
Interaction between IBDV VP3 and IRF7. **(A)** After co-transfecting DF-1 cells with Flag-VP3 and His-IRF7, we harvested samples 48 hours later. Cells were lysed and immunoprecipitation was performed using Flag-tag antibodies, followed by Western blot detection. **(B)** DF-1 cells transfected with p3×FLAG-CMV-14-VP3 and pcDNA3.1-His-IRF7 were subjected to immunofluorescence staining with anti-FLAG rabbit mAb and anti-His mouse mAb.

It has been reported by Ye et al. (2014) that IBDV VP3 can suppress the expression of IFN-β. However, whether IBDV VP3 can interact with chicken IRF7 remains unknown. To investigate this interaction, we co-transfected DF-1 cells with pcDNA3.1-His-IRF7 and p3×FLAG-CMV14-VP3. Following transfection, we performed CO-IP using Flag-tag and His-tag antibodies. The results are shown in **Figure 6A**, the Flag-tag antibodies precipitated Flag-VP3 and concurrently, His-IRF7, demonstrating an interaction between IBDV VP3 protein and IRF7 protein. To further validate the relationship between IRF7 and VP3, an immunofluorescence staining assay was conducted. We observed that exogenous viral protein VP3 colocalized with exogenous IRF7 in the cytoplasm of DF-1 cells (**Figure 6B**).

### 3.7 Overexpression of IBDV VP3 inhibits the expression of IRF7

**Figure 7:**
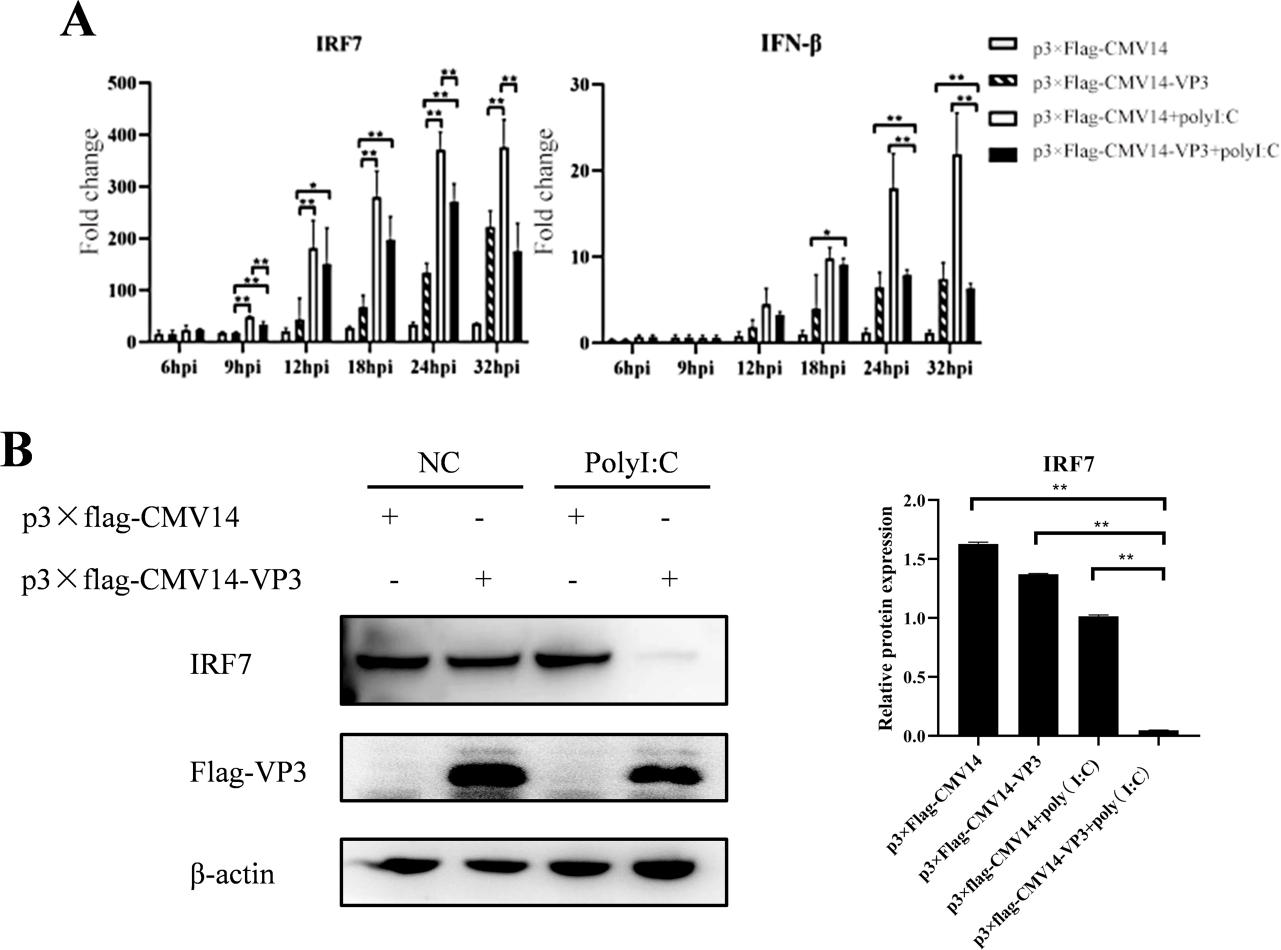
Overexpression of IBDV VP3 inhibits IRF7 expression. **(A)** DF-1 cells were transfected with the p3×FLAG-CMV14-VP3 plasmid for 6 hours and then stimulated with polyI:C. RT-qPCR was used to detect the gene expression levels of IRF7 and IFN-β at various time points post-infection. **(B)** DF-1 cells were transfected with the p3×FLAG-CMV14-VP3 plasmid for 6 hours and then stimulated with polyI:C for 24 h Western blot analysis was performed to detect the protein expression levels of IRF7 and Flag-VP3. Alternatively, relative intensities of IRF7 were normalized to β-actin. Three independent experiments were performed, and the data are presented as the mean ± standard deviation of three replicates from a representative experiment. *p<0.05; **p<0.01.

Since IBDV VP3 interacts with IRF7, we sought to investigate the affecting of IBDV VP3 on IRF7 mRNA and protein levels. DF-1 cells were transfected with either the p3×FLAG-CMV14-VP3 plasmid or the p3×FLAG-CMV14 plasmid, following transfection, the cells were stimulated with polyI:C, an immune activator. Cell samples were collected at various time points post-stimulation, IRF7 and IFN-β expressions were analyzed using RT-qPCR. As shown in **Figure 7A**, compared to the empty vector control group, the mRNA levels of IRF7 and IFN-β in cells transfected with the IBDV VP3 plasmid were significantly reduced after 24 hours of stimulation. Western blot analysis was further used to detect the changes in IRF7 protein levels at 24h post transfection. As shown in **Figure 7B**, the IRF7 protein levels were significantly lower in the IBDV VP3-transfected cells compared to the empty vector control group, consistent with the mRNA level results. These findings indicate that in cells with activated IRF7 signaling, the IBDV VP3 protein reduces both the gene and protein levels of IRF7.

### 3.8 IBDV VP3 promote IRF7 degradation through the proteasome pathway

**Figure 8:**
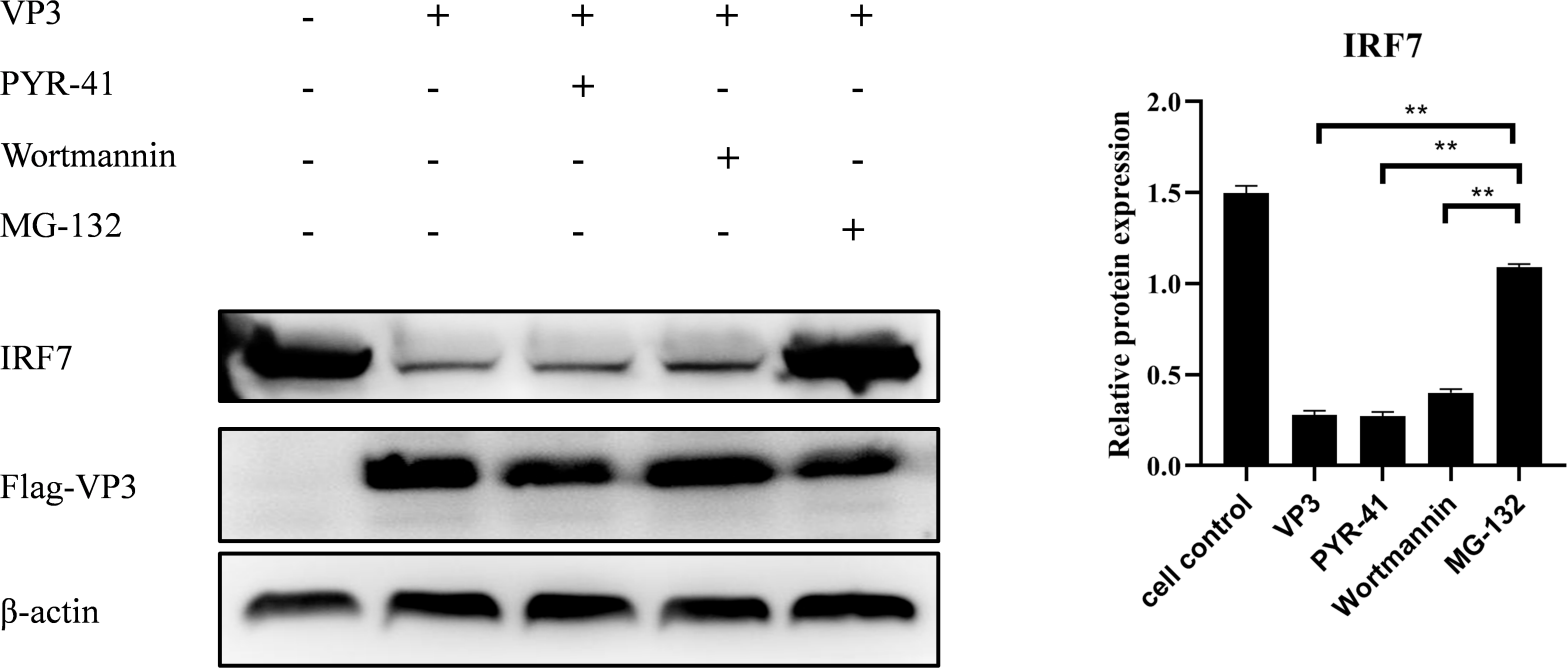
IBDV VP3 promote IRF7 degradation through the proteasome pathway. After transfecting DF-1 cells with the p3×FLAG-CMV14-VP3 plasmid for 6 hours, cells were treated with inhibitor: PYR-41, Wortmannin, or MG132, respectively. Samples were collected 24 h after polyI:C treatment and the IRF7 and VP2 protein levels were analyzed using Western blot. Alternatively, relative intensities of IRF7 were normalized to β-actin. Three independent experiments were conducted, and the data are presented as the mean ± standard deviation of three replicates from a representative experiment. **p < 0.01.

To further explore how IBDV VP3 affects the IRF7 protein level, we transfected DF-1 cells with the p3×FLAG-CMV14-VP3 plasmid. Post-transfection, the cells were treated with MG132, Wortmannin, and PYR-41, respectively. Following this, the cells were stimulated with polyI:C to activate the antiviral immune signaling pathway. After 18 hours, cells were collected for detecting IRF7 protein using Western blot. As shown in **Figure 8**, cells transfected with the p3×FLAG-CMV14-VP3 plasmid showed an increase in IRF7 protein level when treated with the proteasome inhibitor MG132. In contrast, the IRF7 protein was degraded in the other inhibitor treated groups. This indicates that IBDV VP3 protein induces the degradation of IRF7 protein through the proteasome pathway.

## 4 Discussion

In recent years, as the field IBDV strains continue to mutate, an increasing number of gene reassortant strains, gene recombination strains, and Chinese novel variant strains have been confirmed (Fan et al., 2019; Wang et al., 2022a, b), IBDV still poses a threat to the poultry industry worldwide, with traditional vaccines failing to provide full protection (Fan et al., 2020), presenting significant challenges to the control of IBD. IBDV infection represents a complex interplay between the virus and the host, hence understanding the interaction between IBDV and the host can aid in the development of novel vaccines. Since the vital role of IRF7 inducing IFN-α/β antiviral responses both in mammals and chickens (Gao et al., 2019, 2023), it is imperative to investigate the interaction between IBDV and IRF7 to further comprehend the pathogenic mechanism of IBDV.

IRF7 is a crucial transcription factor that regulates the production of IFN-β and located downstream of the TLRs and MDA5 receptors (He et al., 2017; Yu et al., 2022). It has been established that the antiviral activity of the host is usually associated with the elevation of IRF7 and IFN-β levels in the cells after virus infection (Lan et al., 2017). Our study reveals that IBDV triggers an early IRF7-IFN-β response during infection of DF-1 cells. Consistent with this, IBDV, being an RNA virus, has been reported to trigger the TLR3 (He et al., 2017; Quan et al., 2017), TLR7 (Yu et al., 2019) and MDA5 (Chen et al., 2024; Hui and Leung, 2015; Lee et al., 2014; Yu et al., 2022) innate antiviral pathway *in vitro* and/or *in vivo*, which is accompanied by upregulation of IFN-β. Furthermore, IRF7 was also found to be upregulated in chicken thymus at early IBDV infection stages, as demonstrated by both transcriptomic and proteomic analysis (Chen et al., 2024). Taken together, our results confirm that IRF7-IFN-β innate antiviral pathway was activated early after IBDV infection, suggesting that the host cell, including DF-1 cells, attempting to combat IBDV infection by activating the interferon signaling pathway.

Interestingly, we observed that the mRNA levels of IRF7 decreased in DF-1 cells following vvIBDV infection at later time points (≥24 hpi), compared to earlier time points and uninfected controls (**Figure 1A**). Further analysis revealed that the reduction in IRF7 mRNA was dependent on the IBDV titer, with higher titers inducing a more pronounced decrease (**Figure 1C**). However, this trend was not reflected at the protein level. As we observed that the protein levels of IRF7 were significantly reduced during the later stages of infection (≥18 hpi), regardless of the IBDV titer (**Figures 1D–F**). Consistently, the protein level of IFN-β, a downstream factor of IRF7, mirrored the trend of IRF7 (**Figure 1G**). Studies based on integrating proteomics and transcriptomics have shown that transcriptional and protein levels often do not directly correlate (Abdulghani et al., 2019; Chen et al., 2024; Song and Wang, 2019). Current evidence suggests that such discrepancies may result from regulatory mechanisms, including methylation modifications (Cieśla et al., 2023), alternative splicing (Chembazhi et al., 2023), and post-translational modifications (Xu et al., 2021). Therefore, whether the expression of IRF7 in IBDV-infected cells is regulated by these mechanisms requires further investigation. Notably, the trend of IRF7 expression found in the DF-1 cells is consistent with our previous findings from intestinal lamina propria (ILP) cells of chicken infected with vvIBDV which were detected on mRNA level (Chen et al., 2022). All these consistent results suggest that IRF7 transcription is inhibited by IBDV infection. Many viruses have been evolved to block IRF3/7 signaling to inhibit the production of IFN-β, thereby facilitating virus replication within the cells. Examples include Hepatitis C virus (HCV) (Raychoudhuri et al., 2010), Marek’s disease virus (Gao et al., 2019), and avian reovirus (Gao et al., 2023). In Ouyang’s research (Ouyang et al., 2018), attenuation of IRF7 signaling promoted the replication of IBDV in DT40 cells. We also observed that IRF7 knockdown in DF-1 cells enhanced IBDV replication (**Figure 3**), while IRF7 overexpressed inhibited IBDV replication in DF-1 cells (**Figure 4**). Given that type I IFN has been reported to be antagonized after IBDV infection in many studies (He et al., 2017; Lee et al., 2015; Rauf et al., 2011; Smith et al., 2015), it is suggested that IRF7 signaling is suppressed by IBDV, which inhibits IRF7 transcription and protein levels, potentially serving as a strategy for IBDV to evade the host immune system and promote viral replication and dissemination. Taken together, IRF7 is one of important factors that exploited by IBDV to block cell mediated innate antiviral response to facilitate viral replication.

It has been confirmed that upon receiving signals of viral infection, IRFs undergoes phosphorylation, leading to their dimerization and translocation into the cell nucleus, where they binds to the IFN-β promoter and enhances the expression of the IFN-β (Arnold et al., 2013). Many RNA viruses have been shown to regulate the IRF pathway either directly or indirectly, with different viruses exhibited different regulatory mechanisms. For instance, Kaposi’s sarcoma associated herpesvirus (KSHV) blocks the phosphorylation and accumulation of IRF7 during viral infection (Liang et al., 2012), Epstein-Barr virus inhibits the IRF7 dimerization (Wang et al., 2020), SARS coronavirus (SARS-CoV) blocks IRF3 signaling at a step after phosphorylation, but does not inhibit IRF3 dimerization, nuclear localization or DNA binding, the blocking were further confirmed to be related to IRF3 ubiquitination (Matthews et al., 2014). In this study, we observe that IRF7 protein levels, decreased by IBDV infection, were blocked when infected cells were treated with the proteasome inhibitor MG132, suggesting that IRF7 degradation is promoted by IBDV through the proteasome pathway. In 2024, Niu and his peers revealed in a study that IBDV has the power to inhibit the nuclear entry activity of IRF7, thus resulting in the suppression of interferon expression. Taken together, IBDV appears to inhibit the IRF7 signaling by multiple steps, including reducing IRF7 expressing, promoting proteasomal degradation as observed by us and nuclear entry activity reported by Niu et al., 2024. Since IRFs function involves several important critical steps, including phosphorylation, dimerization, nuclear localization or DNA binding, further investigation is required to determine the impact of IBDV infection on other aspects of IRF7 activation.

RNAi technique has emerged as the most desired method for researchers who wish to silence a specific gene of interest and has been extensively used in the scientific researches. RNAi is also an effective antiviral mechanism which include important regulation molecules such as Dicer, Drosha, Exportin5 and Ago2 (Poirier et al., 2021; Shah et al., 2021; van Rij et al., 2006). Mammals express Dicer1 and Ago1 for the biogenesis of both miRNAs and siRNAs but retain the cleavage activity essential for RNAi (Bartel, 2018; Shah et al., 2021). In our study, we only detected IFN-β response to reveal the IRF7 interference on the replication of IBDV. As Shah et al. (2021) concerned that Whether IFN and RNAi contribute in these ways to antiviral immunity in avian remains controversial, since Shah et al. (2021) also found that the expressions of dicer and ago2 were basically blocked in the IBDV infection, further detection of more key genes in the RNAi pathway will be needed to gain a more comprehensive understanding of the interaction mechanism between IBDV and host cells.

IBDV VP3 is the viral protein that has been reported to regulate viral replication through various mechanisms. In 2020, research by Zhang’s group indicated that the IBDV VP3 protein inhibits autophagy during the early stages of IBDV replication (Zhang et al., 2020). Other studies have shown that IBDV VP3 inhibits the host antiviral response, thereby facilitating viral replication and pathogenesis (Busnadiego et al., 2012; Deng et al., 2022). In 2014, Ye and his fellows found that IBDV VP3 can compete with MDA5 to bind intracellular viral genomic dsRNA, suppressing the production of IFN-β. In another study, it was demonstrated that VP3 works synergistically with the chicken RNA binding protein Staufen1 (STAU1) to suppress IFN-β production (Ye et al., 2019). Deng’s group revealed that IBDV VP3 hinders the formation of the TRAF3-TBK1 complex by reducing K33-linked polyubiquitination of lysine 155 on TRAF3, thereby inhibiting MDA5-dependent, IRF3-mediated signaling and ultimately facilitating viral replication (Deng et al., 2021). In this study, we found that IRF7 interacts and colocalizes with IBDV VP3 protein in the cells. Further, we observed that IBDV VP3 protein down-regulates the IRF7 protein production, blocking the proteasome pathway halts IRF7 degradation. These findings are consistent with those observed in DF-1 cells infected with IBDV. Since many viruses have been confirmed to regulate the IRFs signaling by viral proteins (Gao et al., 2023; Liang et al., 2012; Wang et al., 2020), and given our consistent results from both IBDV infection and IBDV-VP3 transfection studies, it can be concluded that IBDV inhibits IRF7 signaling through its VP3 protein to facilitate viral replication. However, the specific mechanism underlying the interaction between VP3 and IRF7 remains unclear. Future research should focus on identifying the key interaction sites of VP3 to elucidate its mechanism of action. Additionally, it is worth investigating whether VP3 affects the stability and function of IRF7 through other pathways, such as phosphorylation, dimerization, or ubiquitination modifications. Animal studies are also needed to assess the impact of VP3 protein on IBDV pathogenicity, immune response and disease outcome. These investigations will help evaluate the feasibility and effectiveness of targeting the VP3 protein for antiviral therapy. Collectively, these studies could provide deeper insights into the molecular mechanisms involved and potentially facilitate the development of antiviral drugs aimed at the VP3 protein.

It is worth noted that infections with different virulent strains of IBDV differing in their impact on IRF7 expression was observed in our study. In DF-1 cells infected with vvIBDV, IRF7 was initially upregulated and then degraded. Conversely, in cells infected with the attenuated vaccine strain B87, IRF7 gene expression levels did not significantly change at various time points, nor did IFN-β levels. We only observed a transient upregulation of IFN-β at 18 hpi in B87-infected DF-1 cells which is consistent with our previous research, that study showed transient activation of IFN-β antiviral response at 4 hpi in the Gut-associated lymphoid tissues (GALT) cells isolated from B87-infected chickens (Chen et al., 2022). The differential effect of different virulent IBDV on antiviral pathways have been noted by many researchers. Our group demonstrated that infection with the intermediate virulent strain NN040124 and vvIBDV strain NN1172 showed a down-regulating effect on IFN-β expression in chicken peripheral blood mononuclear cells and three-week-old chickens, respectively (He et al., 2017). In contrast, B87 infection showed initially downregulated and later upregulated IFN-β levels in chickens. However, in 2006, Eldaghayes research group found no significant change in IFN-β mRNA expression after infection of SPF chicken with classical strain F52/70 (Eldaghayes et al., 2006). In contrast, UK661 infection initially down-regulated but later recovered to baseline. These results indicate that B87 infection does not significantly affect the innate antiviral response, including the IRF7 signaling step, which might be related to the easier replication and dissemination of B87, and consistent with the biology of this vaccine strain as we concerned (Chen et al., 2022). This result further confirms the crucial role of the IRF7 signaling in the pathogenicity of IBDV.

In summary, we demonstrated for the first time that IBDV antagonizes host IFN-β by regulating the IRF7 signaling via IBDV VP3 protein. This regulation involves down regulating IRF7 expression, promoting IRF7 degradation through the proteasome pathway, thereby facilitating viral replication within the cells. Different virulent strains of IBDV exhibit varying regulatory effects on the cellular IRF7 signaling pathway, with vvIBDV has a significant effect and attenuated strain has no effect. These findings may expand our current knowledge about the mechanisms that IBDV exploits to evade host antiviral innate immunity and promote the development of more effective strategies for prevention and control of IBD in clinical practice.

## Data Availability

The raw data supporting the conclusions of this article will be made available by the authors, without undue reservation.
